# Suicide Among the Emergency Medical Systems Occupation in the United States

**DOI:** 10.5811/westjem.2020.10.48742

**Published:** 2021-01-20

**Authors:** Neil H. Vigil, Samuel Beger, Kevin S. Gochenour, Weston H. Frazier, Tyler F. Vadeboncoeur, Bentley J. Bobrow

**Affiliations:** * University of Arizona College of Medicine-Phoenix, Department of Emergency Medicine, Phoenix, Arizona; † Mayo Clinic Florida, Department of Emergency Medicine, Jacksonville, Florida; ‡ University of Texas Health Sciences Center of Houston, Department of Emergency Medicine, Houston, Texas

## Abstract

**Introduction:**

Suicide claimed 47,173 lives in 2017 and is the second leading cause of death for individuals 15–34 years old. In 2017, rates of suicide in the United States (US) were double the rates of homicide. Despite significant research funding toward suicide prevention, rates of suicide have increased 38% from 2009 to 2017. Recent data suggests that emergency medical services (EMS) workers are at a higher risk of suicidal ideation and suicide attempts compared to the general public. The objective of this study was to determine the proportionate mortality ratio (PMR) of suicide among firefighters and emergency medical technicians (EMT) compared to the general US working population.

**Methods:**

We analyzed over five million adult decedent death records from the National Occupational Mortality Surveillance database for 26 states over a 10-year non-consecutive period including 1999, 2003–2004, and 2007–2013. Categorizing firefighters and EMTs by census industry and occupation code lists, we used the underlying cause of death to calculate the PMRs compared to the general US decedent population with a recorded occupation.

**Results:**

Overall, 298 firefighter and 84 EMT suicides were identified in our study. Firefighters died in significantly greater proportion from suicide compared to the US.working population with a PMR of 172 (95% confidence interval [CI], 153–193, P<0.01). EMTs also died from suicide in greater proportion with an elevated PMR of 124 (95% CI, 99–153), but this did not reach statistical significance. Among all subgroups, firefighters ages 65–90 were found to have the highest PMR of 234 (95% CI, 186–290), P<0.01) while the highest among EMTs was in the age group 18–64 with a PMR of 126 (95% CI, 100–156, P<0.05).

**Conclusion:**

In this multi-state study, we found that firefighters and EMTs had significantly higher proportionate mortality ratios for suicide compared to the general US working population. Firefighters ages 65–90 had a PMR more than double that of the general working population. Development of a more robust database is needed to identify EMS workers at greatest risk of suicide during their career and lifetime.

## INTRODUCTION

Suicide is a leading cause of death in the United States (US), claiming the lives of over 47,000 Americans in 2017. 1 Suicide is the 10th leading cause of death for all ages in the US and the second leading cause of death for people ages 15–34. In 2017, rates of suicide in the US were double the rates of homicide. 1 In an attempt to address this public health problem, the National Institutes of Health increased funding for suicide prevention from $39 million in 2008 to $103 million in 2017. 1 Despite these efforts, rates of suicide have increased 38% since 1999 from 10.48 per 100,000 to 14.48 per 100,000 in 2017. 1

In an attempt to address increasing suicide rates in the US, researchers have sought to identify leading risk factors of suicide as well as populations at greatest risk. 1–6 National surveys suggest that emergency medical services (EMS) workers, including firefighters and emergency medical technicians (EMT), are at higher risk of experiencing suicidal ideation and suicide attempts compared to the general public.^7^–^10^ These elevated levels of suicidal ideation and suicide attempts are hypothesized to be the result of the occupational hazards associated with the EMS profession, which include routine exposure to high levels of physical and psychological stress. 2,3,11

While several studies have quantified individual risk factors among EMS workers, there is scant published research on completed suicide in this population. We analyzed the National Occupational Mortality Surveillance (NOMS) database to examine the proportion of death by suicide among firefighters and EMTs compared to other US decedents with a recorded occupation.

## METHODS

### Study Design

This was a retrospective study of 10 years of mortality data from the NOMS database. The NOMS database is maintained by the National Institute for Occupational Safety and Health (NIOSH) and is used to periodically monitor causes of death across occupations and industries to facilitate occupational mortality surveillance over time. 12 The database collects mortality records for decedents ages 18–90 with a recorded occupation. The dataset used in our analysis includes 5,070,335 adults, ages 18–90, whose records of death were collected from state-level vital statistics offices over 10 non-consecutive years during 1999, 2003–2004, and 2007–2013. 12

We used proportionate mortality ratio (PMR) analysis to determine the pattern of suicide by occupation.^13^ A PMR indicates whether a proportion of deaths due to a specific cause is high or low for a particular population and therefore approximates the death rate. We chose the PMR to assess risk for this study instead of other estimations of risk due to the difficulty in accurately estimating the at-risk population for a given year based on job code. The PMR Query System calculates PMRs by occupation by comparing the proportion of deaths from a specific cause within a specific occupation with the proportion of deaths due to that cause across all occupations (multiplied by 100). This can be further stratified by age, race, and gender. A PMR of greater than 100 is considered elevated over all other occupations combined. 12 A regulatory determination that this study was not human subjects research, as defined by 45 CFR 46.102(f), was approved by the University of Arizona Human Subjects Protection Program institutional review board.

**Population Health Research Capsule**

What do we already know about this issue?

Emergency medical services (EMS) workers have a higher risk of suicidal ideation and attempts compared to the general public, possibly the result of occupational exposures.

What was the research question?

Do EMS workers commit suicide at a higher proportion compared to the general US working population?

What was the major finding of the study?

Suicide among EMS workers was proportionately higher than the general US working population.

How does this improve population health?

Identifying those at highest risk of suicide during their lifetime is a critical step in developing crucial prevention strategies and resources.

### Study Setting and Population

A total of 26 states contributed mortality data to the NOMS dataset used in our analysis. Death certificates were completed by funeral directors and medical certifiers and contained unique fields including the cause of death, usual occupation, and demographic information. Underlying cause of death mortality data were coded utsing the International Classification of Diseases, 10^th^ Edition (ICD–10). 14 We collected records of decedents with a known occupation from Colorado, Florida, Georgia, Hawaii, Idaho, Indiana, Kansas, Kentucky, Louisiana, Michigan, North Carolina, North Dakota, Nebraska, New Hampshire, New Jersey, New Mexico, Nevada, Ohio, Rhode Island, South Carolina, Texas, Utah, Vermont, Washington, Wisconsin, and West Virginia. The population at risk includes all men and women ages 18–90 with a known occupation who were at risk of dying over the study period. Access to PMRs, methods, and further information is available at https://www.cdc.gov/niosh/topics/noms. 15

### Data Collection and Processing

We collected NOMS data from the NOMS electronic PMR Query System.^16^ The NOMS dataset included age, race, gender, underlying cause of death, and occupation. EMS occupations used in this study included firefighters and EMTs. EMT deaths were inclusive of paramedic death records in accordance with current US Centers for Disease Control and Prevention occupational coding. Occupation fields were coded using the NIOSH 1990 or 2000 census industry occupation code lists based on the year of death. Firefighters were categorized using the 1990 census occupation codes 413 (supervisors, firefighting, and fire prevention occupations) and 417 (firefighting occupations) or 2000 census occupation codes 372 (first-line supervisors/managers of firefighting and prevention workers) and 374 (firefighters).

The 2000 census established occupation code 340 (EMTs and paramedics), which was used to categorize EMTs and paramedics in this study. 12 Prior to the 2000 census, EMTs and paramedic deaths were not recorded with a unique occupation code. Due to the inability to identify EMT deaths prior to the incorporation of the 2000 census industry and occupation code list, our study did not include EMT deaths prior to the year 2000. For this study, suicide was defined as ICD-10 codes X60–X84 and Y87.0.^14^ We excluded decedents who were students, volunteers, unemployed, or had an unknown occupation or industry.

### Data Analysis

We calculated PMRs for groups stratified by race (White, Black, all races combined), age (18–64, 65–90, 18–90), and gender (male, female) using the PMR Query System developed by NIOSH. 17 PMRs are calculated when the total population at risk is not known and rates of death or standardized mortality ratios (SMR) cannot be calculated. 18 A rate of death or SMR could not be calculated for this occupation-based analysis due to the total number of workers in EMS being unknown. We calculated 95% confidence intervals (CI) for the observed PMRs. If the observed number of deaths for an occupation was 1000 or less, we calculated the 95% CI based on the Poisson distribution, while for occupations with greater than 1000 deaths CIs were calculated using the Mantel and Haenszel chi-square test.^19^,^20^ Due to confidentiality agreements with the reporting states, the number of deaths are reported in tables as “<5,” when a cell is based on fewer than five deaths.

## RESULTS

There were 5,070,335 deaths entered into the NOMS database during the study period. Of those deaths, there were 298 firefighter and 84 EMT deaths attributed to suicide (Table 1). The PMR for firefighters ages 18–90 was 172 (95% CI, 153–193, *P*<0.01] compared to the general US working population (Table 2). When stratified for age the PMR for firefighters 18–64 years old was 157 (95% CI, 136–179), *P*<0.01), and 65–90 years old was 234 (95% CI, 186–290, *P*<0.01) (Table 2). A trend toward elevated PMR for EMTs was observed compared to the general US working population with a PMR of 124 (95% CI, 99–153), however, this trend did not reach statistical significance (Table 3). When stratified for age, the PMR for EMTs 18–64 years old was 126 (95% CI, 100–156, *P*<0.05) (Table 3). PMR for EMTs 65–90 years old could not be calculated due to confidentiality agreements with suicides <5. The PMR for White male firefighters ages 18–90 was 130 (95% CI, 114–147, *P*<0.01], ages 18–64 was 126 (95% CI, 108–146, *P*<0.01], and ages 65–90 was 139 (95% CI, 111–173, *P*<0.01]) (Table 2).

## DISCUSSION

Our study identified a significantly higher proportion of completed suicides in firefighters ages 18–90 and EMTs ages 18–64 compared to the general US working population. Although there is previous research showing increased firefighter and EMT risk for suicidal ideation, this is the first multi-state study to our knowledge suggesting a higher rate of completed suicide for EMTs and firefighters.

While there are multiple studies examining law enforcement suicide, there is a paucity of data regarding this topic in firefighters and EMTs.^21^–^23^ Those studies available suggest that firefighters have an increased prevalence of suicidal ideation, plans, and attempts (46.8%, 19.2%, and 15.5%,) compared to the general population (13.5%, 3.9%, and 4.6%). 7,22,24 Despite increased suicide risk factors, five previous mortality studies found a decreased SMR for suicide among firefighters during the period 1915–1999, which is in contrast to our results. 25–29 The era in which the mortality data for these studies were collected may provide insight into our differing conclusions. Vigil et al (2018) identified changes to the role of a firefighter from fire suppression to emergency medical aid in the later 20th century, with the development of the modern-day EMS system.^2^ In the modern-day EMS system firefighters are often dispatched as the closest available first responder in addition to a transport-capable EMS unit, and frequently arrive up to several minutes prior to a transport-capable EMS unit.^30^ From 1999 to 2013 fire calls decreased nationally by 31% from 1,823,000 to 1,240,000, while medical aid calls have increased by 198% from 11,484,000 to 22,750,500.^31^

Although studies prior to 1999 do not show elevated suicide mortality ratios among firefighters, a more recent study from Arizona found that EMS providers are significantly more likely to die from suicide than the general population. 2 EMTs had an odds ratio of 1.39 for suicide over a seven-year period from 2009–2015.^2^ While this was a single-state study, the results are consistent with our findings on the national level.

EMS personnel are exposed to many stressors and traumatic events that have been shown to place them at greater risk for mental health disorders and suicidal behavior.^32^,^33^ These often comorbid risk factors include alcohol use, sleep disturbances, post-traumatic stress, and chronic exposure to stress in the work-place.^34^–^38^ An important modifiable stressor that members of EMS regularly encounter is chronic sleep deprivation, which has been found to increase rates of suicide.^39^–^41^ Chronic sleep deprivation among EMS workers is common, and workers are required to respond to urgent calls disrupting normal sleep patterns.^42^–^44^ Sleep deprivation has also been shown to exacerbate comorbid risk factors for suicide such as post-traumatic stress disorder (PTSD) and depression, which is prevalent among EMS workers.^45^,^46^ Those with PTSD have reported sleep disturbances as high as 91% and nightmares as high as 71%.^47^

Furthermore, alcohol abuse may play a role in the elevated risk of suicide among EMS providers. Researchers hypothesize that firefighters may drink excessive amounts of alcohol in an attempt to suppress the symptoms of PTSD.^50^ In a survey of 656 firefighters, more than 50% reported recent heavy or binge drinking, while 9% reported driving while intoxicated.^51^,^52^ The combination of alcohol and PTSD significantly increases the likelihood of suicidal behavior.^50^

Repeated exposures to traumatic events may also place EMS providers at increased risk of suicide. In a recent survey of 1789 EMS workers, 69% reported experiencing violence directed at them in the prior 12 months.^48^ Of particular importance, exposure to suicides has been shown to independently increase the risk of suicidal ideation.^49^ Kimbrel et al (2016) found that 100% of firefighter respondents reported at least one suicide exposure, and found that firefighters with 12 or more suicide exposures had a lifetime suicidal ideation rate of 61.1% compared to 31.6% for those with 11 or fewer.^49^ Additionally, stressful situations with a low threshold for failure have been proposed as a cause for increased suicide rates in EMS workers.^37^ These situations place firefighters and EMTs at risk for increased rates of anxiety and depression, both of which have been implicated in increased risk for suicide.^36^

Our findings, combined with the multiple suicide risk factors previously found among EMS workers, highlights the urgent need for further research among this at-risk cohort. In addition to further exploration of suicide in EMS workers, focused investigation of retirement age (65–90) firefighters is needed. Our study’s finding of a PMR of 234 within this subgroup may indicate that the elevated risk of suicide in this occupation may extend far beyond the time one leaves the job. Additionally, EMS workers may benefit from identification and implementation of effective interventions to reduce the risk of suicide.

## LIMITATIONS

We have identified several limitations in our study. First, the use of PMRs are susceptible to biases including regression to the null, “Healthy Worker Effect,” and over- or under-representation of mortality from other causes of death. 23,53,54 Our study used the PMR because the total at-risk (currently living) population of firefighters and EMTs was unknown.

There are limitations when working with census data and occupational death reporting. As we stated above, EMT and paramedic job codes were not included on occupational death certificates prior to 2000, this could be a reason that the PMR for EMT suicide we observed did not achieve statistical significance. Misclassification of occupation may have occurred because information on death certificates was recorded by funeral directors and medical certifiers. However, Petersen et al (1974) reported an 80% accuracy of occupations listed on death certificates compared to surviving family-member interviews. 55,56 Death records assign a single occupation to each decedent, potentially under-representing EMTs and firefighters with second careers. Suicides may have been misclassified as a non-suicide resulting in fewer reported suicides as has been demonstrated with suicide among police officers.^57^ A final possible confounding factor with occupational death reporting of this nature is that the decedent’s place of residence may not accurately represent the same locality as their place of work.

## CONCLUSION

In this multi-state study, firefighters and EMTs had significantly higher proportionate mortality ratios for suicide compared to the general US working population. Firefighters ages 65–90 had a PMR more than double that of the general working population. Development of a more robust database is needed to identify EMS workers at greatest risk of suicide during their career and lifetime.

## Figures and Tables

**Table 1:** Suicide in US working firefighters and emergency medical technicians ages 18–90 NIOSH surveillance, 1999, 2003–2004, 2007–2013.

Age group	Firefighter suicides	EMT suicides
18–90	298	84
18–64	215	83
65–90	83	<5*

* Due to confidentiality agreements with states, the number of deaths are reported in tables as “<5” when a cell is based on less than five deaths.

*NIOSH*, National Institute for Occupational Safety and Health; *EMT*, emergency medical technician.

**Table 2:** Proportionate Mortality Ratios (PMR)^A^ for Suicide: Firefighters by age, gender, and race ages 18–90, vs. U.S. working population NIOSH National Occupational Mortality Surveillance (NOMS), 1999, 2003–2004, 2007–2013.

	Suicides	PMR	95% CI
Firefighters			
Age group			
18–90 years old	298	172**	153–193
18–64 years old	215	157**	136–179
65–90 years old	83	234**	186–290
White males			
18–90 years old	258	130**	114–147
18–64 years old	177	126**	108–146
65–90 years old	81	139**	111–173
Black males			
18–90 years old	8	160	69–316
18–64 years old	8	177	77–349
65–90 years old	<5^B^	–	–
White females			
18–90 years old	8	175	76–345
18–64 years old	8	184	80–363
65–90 years old	<5^B^	–	–
Black females			
18–90 years old	<5^B^	–	–
18–64 years old	<5^B^	–	–
65–90 years old	<5^B^	–	–

A. A PMR greater than 100 is considered elevated over the average compared to the general United State’s working population.

B. Due to confidentiality agreements with states, the number of deaths are reported in tables as ‘<5’ when a cell is based on less than 5 deaths, making the exact calculation of death in that category impossible.

* indicates a significance (P-value) < 0.05

** indicates a significance (P-value) < 0.01

*PMR*, proportionate mortality ratio; *CI*, confidence interval.

**Table 3:** Proportionate Mortality Ratios (PMR)^A^ for Suicide: EMTs by age, gender, and race Ages 18–90, vs. U.S. working population NIOSH National Occupational Mortality Surveillance (NOMS), 1999, 2003–2004, 2007–2013.

	Suicides	PMR	95% CI
EMTs			
Age Group			
18–90 years old	84	124	99–153
18–64 years old	83	126*	100–156
65–90 years old	<5^B^	–	–
White Males			
18–90 years old	62	102	78–131
18–64 years old	61	103	79–133
65–90 years old	<5^B^	–	–
Black Males			
18–90 years old	<5^B^	–	–
18–64 years old	<5^B^	–	–
65–90 years old	<5^B^	–	–
White Females			
18–90 years old	17	132	77–212
18–64 years old	17	135	79–217
65–90 years old	<5^B^	–	–
Black Females			
18–90 years old	<5^B^	–	–
18–64 years old	<5^B^	–	–
65–90 years old	<5^B^	–	–

A. A PMR greater than 100 is considered elevated over the average compared to the general United State’s working population.

B. Due to confidentiality agreements with states, the number of deaths are reported in tables as ‘<5’ when a cell is based on less than 5 deaths, making the exact calculation of death in that category impossible.

* indicates a significance (P-value) < 0.05

** indicates a significance (P-value) < 0.01

*PMR*, proportionate mortality ratio; *CI*, confidence interval.
